# Mental distress, alcohol use and help-seeking among medical and business students: a cross-sectional comparative study

**DOI:** 10.1186/1472-6920-11-92

**Published:** 2011-11-07

**Authors:** Marie Dahlin, Caroline Nilsson, Emelie Stotzer, Bo Runeson

**Affiliations:** 1 Department of Clinical Neuroscience, Centre of Psychiatric Research, St. Goran, Karolinska Institutet, Stockholm, Sweden; 2 Sundsvalls Sjukhus [Sundsvall's Hospital], Sundsvall, Sweden; 3 Department of Women's Health, Sodersjukhuset, Stockholm, Sweden

## Abstract

**Background:**

Stress and distress among medical students are thoroughly studied and presumed to be particularly high, but comparative studies including other student groups are rare.

**Methods:**

A web-based survey was distributed to 500 medical students and 500 business students. We compared levels of study stress (HESI), burnout (OLBI), alcohol habits (AUDIT) and depression (MDI), and analysed their relationship with self-assessed mental health problems by logistic regression, with respect to gender.

**Results:**

Medical students' response rate was 81.6% and that of business students 69.4%. Business students scored higher on several study stress factors and on disengagement. Depression (OR 0.61, CI_95 _0.37;0.98) and harmful alcohol use (OR 0.55, CI_95 _0.37; 0.75) were both less common among medical students. However, harmful alcohol use was highly prevalent among male students in both groups (medical students 28.0%, business students 35.4%), and among female business students (25.0%). Mental health problems in need of treatment were equally common in both groups; 22.1% and 19.3%, respectively, and was associated with female sex (OR 2.01, CI_95 _1.32;3.04), exhaustion (OR 2.56, CI_95 _1.60;4.10), lower commitment to studies (OR 1.95, CI_95 _1.09;3.51) and financial concerns (OR 1.81 CI_95 _1.18;2.80)

**Conclusions:**

Medical students may not be more stressed than other high achieving student populations. The more cohesive structure of medical school and a higher awareness of a healthy lifestyle may be beneficial factors.

## Background

Medical students are thoroughly investigated with regard to stress and mental health, and often suggested to be especially subject to stress and also depression [1-3]. Along with these findings, it has been argued that medical education is particularly stressful, but also that medical students carry certain personality traits such as perfectionism or performance-based self-esteem, that make them vulnerable to mental distress [4,5]. We have previously shown that self-rated depression is more common among Swedish medical students than in the general population [3]. Further, some studies report more distress among female medical students than their male peers [3,6], which is in accordance with a higher depression rate for women in the general population. Mental health among young people is declining, shown by e.g. increased use of psychiatric emergency care and psychotropic medication [7,8]; and higher education may be a risk factor in itself [9]. Comparisons of medical students with other university student populations are surprisingly rare, but a few can be traced. A British study showed that medical students had no higher levels of health-anxiety and worry than students of English and Law [10], and a Canadian study found that medical students were less distressed than undergraduate law students and graduate students, respectively [11]. Two studies, from Sweden and Turkey respectively, have reported higher levels of distress among medical students compared to other university student populations [6,12].

Further, medical students have been suggested to be reluctant to seek help and (as is also true for medical professionals [13]) tend to by-pass the "regular" health-care system or avoid formal consultations [14,15]. We have only found one comparative study of students, where both psychology students and medical students reported worries about consulting a doctor for psychological problems, with regard to possible future professional dealings with that person. Medical students, in contrast to those studying psychology, had the same concerns if they were to seek help for somatic complaints [16].

We wanted to evaluate medical students' distress and mental problems in need of treatment against another student population with presumed comparative levels of study demands. In this cross-sectional survey, we compared study stress, burnout levels and mental health indicators as well as help-seeking behaviour between students at the medical school at Karolinska Institutet (KI), and the business programme at Stockholm School of Economics (SSE), both Stockholm, Sweden. Considering previous findings on medical students, we also wanted to address possible gender effects. The choice of comparison sample was based on the assumption that the groups were fairly homogenous and that the two universities attract students of similar characteristics. There is hard competition for admission to both programmes and high status attached to the medical profession as well as the expected careers of the SSE students, and both universities are considered highly prestigious. The gender distribution also corresponds fairly well, all of which supports the validity of this comparative study.

## Methods

### Setting

Higher education in Sweden is tuition free. All students are also entitled to general financial aid, composed of loans and minor grants. The student financial aid is limited to six years, which may cause students problems at the end of long programmes.

At Karolinska Institutet about 130 students are admitted twice a year to enter the medical programme of 5.5 years. All Swedish universities admit students in national competition based on upper secondary school marks or the Swedish Scholastic Assessment Test (SweSAT), a general knowledge test focusing on mathematics as well as Swedish and English comprehension. Besides these traditional ways of admittance, at KI two thirds of the positions are filled through selection from a cognitive test and interviews conducted locally. The curriculum is fixed and the students thus follow a set course of studies. At the time of the study, the curriculum followed a traditional Flexnerian model, with set courses in pre-clinical (semesters 1-5) and clinical stages (semesters 6-11). There are no graded marks at any stages of medical school in Sweden, only pass or fail, for preclinical and clinical courses alike and whether examined by written tests, OSCE's or as an evaluation of clinical rotation.

Stockholm School of Economics is a private university with an annual intake of around 300 students entering a four-year Master of Science in Economics and Business. Admission for 75% of the students at SSE is based on the upper secondary school marks, and 15% on the SweSAT. The final 10% are admitted on distinguished achievements. During the first two years, the basic stage, all of the students take the same courses in Basic Economics and Business Administration, but by choice of order. These years are followed by two years of specialisation. During the basic stage, teaching methods are largely based on didactic lectures, but change to more project-based learning in the last two years. Unlike KI, SSE at the time of the study had a four-grade marking scale.

### Subjects

The study population consisted of all students enrolled at the business programme of Stockholm School of Economics (SSE), approximately 1100, and the medical programme at Karolinska Institutet (KI), Stockholm, approximately 1400, in the autumn semester of 2006. A total of 1000 students were randomly drawn from the universities' registers of active students of all stages in October 2006, 500 from each site, in order to obtain samples of similar size from both schools. The samples were recruited from early (preclinical/basic) and late (clinical/advanced) stages of the curriculum, by an equal number of 250 students. No formal power estimation was performed, but the sample size was assumed to reveal group differences over site and sex with regard to depression, with an expected point prevalence of 5%. E-mail addresses and postal addresses were available. Seven students from SSE were excluded, three since they had terminated their studies, three studied elsewhere and one was unreachable via e-mail or mail.

The study was conducted from November until the end of December 2006 when no general examination periods were held at SSE, since such periods are not employed at KI. We sent e-mails to the included students, containing information about the study and a link to a web-based questionnaire (Websurvey™ software), which could only be filled out if subjects had ticked a box giving their informed consent. The software provided identified information on response status, but yielded a response database that was entirely anonymised. We sent out three reminders to the KI students and four to the SSE students to improve the difference in response rate. In addition, a letter with a paper questionnaire was sent by ordinary mail to 179 SSE students who had not replied. All respondents received a cinema voucher.

The Board of Research Ethics at Karolinska Institutet assessed the study and judged that no formal ethical approval was required according to Swedish regulations. Ethical principles were adhered to. The invited students were informed that participation in the study was entirely optional and there was no possibility to identify responders of the electronic survey. The responses of the questionnaires sent in paper-format were registered upon reception and subsequently entered anonymously into the data set.

### Measures

Respondents were asked to fill out gender, age, cohabiting status, whether they had children and sources of financial support. Paid employment outside studies was recorded as either "No", "Occasional days per month", "Yes, half-time" (meaning 20 hours/week in Sweden) or "Yes, more than half-time".

Study stress was assessed by the Higher Education Stress Inventory (HESI), comprising 33 items on study conditions, rated on a four-point Likert type scale [3]. A factor analysis revealed 9 factors, together comprising 31 of the items and explaining 41% of the variance. Of these, 7 were used in the study: negative psychosocial climate (Cronbach's α 0.73), worries about future endurance/capacity (WFEC, Cronbach's α 0.82), insufficient feedback (Cronbach's α 0.66), low commitment (Cronbach's α 0.67), academic workload (Cronbach's α 0.67), role conflict (Cronbach's α 0.58) and financial concern (Cronbach's 0.57), explaining 36% of the variance. The excluded factors had very low Cronbach's α's (below 0.38).

We measured the burnout dimensions exhaustion (Cronbach's α 0.81) and disengagement (Cronbach's α 0.78) by the Oldenburg Burnout Inventory (OLBI), adapted for students [5,17]. Each dimension is computed as means of the pertaining 8 items, scored on a four-point Likert scale. For the HESI and the OLBI alike, each factor can take on values between 1 and 4, where higher scores indicate more distress. Each factor was also dichotomised by median split. Cronbach's α's given above are all computed on this dataset.

Depressive symptoms were assessed by the Major Depression Inventory; a tool validated for screening and diagnostic purposes in general population samples. A score above 27 has been suggested as indicative of a clinically significant depression [18]. Alcohol habits were screened by the Alcohol Use Disorder Identification Test (AUDIT); we defined harmful use as a sum score equal to or above 11, and weekly drinking to intoxication as scoring equal to or above 3 on the third item [19,20].

The respondents were asked to indicate if they assessed themselves as having had mental health problems since entering their studies, whether they considered these problems to be in need of treatment (MHPT), and if and where they had applied for professional help. This measure has been used previously in studies of Norwegian medical students and interns [21].

### Statistics

Since several measures were not normally distributed, we used the Mann-Whitney U-test for comparisons of ratings for stress and burnout, stratifying by sex (except for analyses of stage of education). Odds ratios were computed for proportions, controlling for sex, also enabling estimations of its effect. We used bivariate logistic regression to compute differences in depression and alcohol use between the two subsamples, controlling for sex. Additional analyses, where curricular stage was included as an independent variable, were also performed. Finally, multivariable logistic regression analysis was carried out for assessing the relationships between mental health problems in need of treatment and study stress, burnout and weekly drinking to intoxication. For the latter, the study stress and burnout variables were dichotomised by median split.

## Results

The response rate was 81.6% at KI (408) and 69.3% (342) at SSE. The medical students were slightly older; median 24 yrs (Inter Quartile Range, IQR, 22-27) than the business students, 23 yrs (IQR 22-25). At KI, 61.5% of responders were women, compared to 42.1% at SSE, reflecting the current proportions of the two programmes. Women were more prone to respond than men at both sites. Further, medical students more commonly lived with a partner and had children than business students. Business students more commonly received economic support from their parents and worked during the semester (Table 1). They also worked longer hours; 17% (n = 59) worked 20 hours/week or more, compared to 5% (n = 20) of the medical students (OR 4.04, CI_95 _2.38; 6.87).

**Table 1:** Demographic data of responders

	KI, Medical students		SSE, Business students			
	n = 408		n = 342			
	%	n	%	n	OR	CI_95_
Women	63.5	251	36.5	144	2.20	1.64;2.95
Do not receive student funding	17.5	70	22.5	76	0.71	0.50;1.02
Paid employment during semester	49.5	202	62.0	211	0.60	0.45;0.81
Economic support from parents	25.0	102	36.8	125	0.57	0.42;0.78
Married, partnership, partner	32.6	133	21.3	73	1.78	1.28;2.48
With children	9.1	37	2.3	8	4.16	1.91;9.07

KI = Karolinska Institutet, SSE = Stockholm School of Economics. Odds ratios are computed for group differences.

### Study stress and burnout

In Table 2 results on study stress and burnout are shown. Business students of both sexes rated significantly higher than medical students on Negative psychosocial climate, Low commitment and Insufficient feedback. Insufficient feedback was the most highly rated stress factor at both sites. Low commitment and Role conflict received low scores in both samples.

**Table 2:** Descriptive data on study stress and burnout, stratified by sex.

	KI, Medical students				SSE, Business students							
	Females		Males		Females		Males		U-tests			
	Med	IQR	Med	IQR	Med	IQR	Med	IQR	**z**_**fem**_	**p**_**fem**_	**z**_**male**_	**p**_**male**_
Negative psychosocial climate	1.80	(1.40-2.20)	1.60	(1.40-2.00)	2.00	(1.60-2.40)	2.00	(1.60-2.40)	-5.05	<.001	-5.74	<.001
Worries about future endurance/capacity	2.50	(2.00-3.00)	2.00	(1.50-3.00)	3.00	(2.00-3.00)	2.50	(1.50-3.00)	-0.21	.837	-0.28	.778
Insufficient feedback	2.67	(2.00-3.00)	2.67	(2.00-3.00)	3.00	(2.33-3.33)	2.67	(2.33-3.33)	-3.23	.001	-3.64	<.001
Low commitment	1.33	(1.00-1.67)	1.33	(1.00-1.67)	1.67	(1.33-2.00)	1.67	(1.33-2.00)	-4.95	<.001	-5.17	<.001
Academic workload	2.33	(2.00-2.67)	2.33	(1.67-2.67)	2.67	(2.00-3.00)	2.33	(1.67-2.67)	-4.30	<.001	-0.01	.998
Role conflict	1.33	(1.00-2.00)	1.33	(1.00-1.67)	1.33	(1.08-1.67)	1.33	(1.00-1.67)	-0.83	.409	-0.56	.573
Financial concerns	2.00	(1.50-2.50)	2.00	(1.50-2. 50)	2.00	(1.50-2.87)	1.75	(1.00-2.50)	-0.53	.596	-2.89	.004
Disengagement	1.87	(1.50-2.25)	2.00	(1.75-2.38)	2.19	(1.74-2.63)	2.25	(2.00-2.62)	-5.51	<.001	-4.35	<.001
Exhaustion	2.37	(2.00-2.87)	2.25	(2.00-2.50)	2.63	(2.13-2.87)	2.25	(1.87-2.75)	-1.98	.048	-0.71	.476

Mann-Whitney U-test. U-tests were performed stratified by sex.

KI = Karolinska Institutet; SSE = Stockholm School of Economics; Med = Median; IQR = Interquartile range

Medians are derived from factor means of Likert type response scales, range 1-4.

Academic workload was rated higher among female business students than female medical students. At both universities female students scored higher than men on the stress factor Worries about future endurance (WFEC). Business students reported more disengagement than medical students. Exhaustion was not significantly different between the universities, but ranked as a more prominent problem than disengagement in both samples.

For medical students, WFEC (Median 2.5 [IQR 2.0;3,0] vs. 3.0 [2.0;3.5], p < 0.0001) and Role Conflict (1.3 [1.3;1.7] vs. 1.3 [1.0;2.0], p = 0.032) were rated higher in the clinical phase, whereas Workload (2.3 [2.0;3.0] vs. 2.0 [1.7;2.3], p < 0.0001) was higher in the preclinical phase. Business students at the advanced stage were less concerned with Low feedback (3.0 [2.6;3.3] vs 2.7 [2.3;3,0], p = 0.003) than those in the basic years (data on curricular stage not shown in table). In addition, business students at the basic stage rated Exhaustion higher than those at the advanced stage (2.5 [2.19;2.9] vs. 2.4 [1,9;2.8. p = 0.030) (data not shown in table).

### Depression and alcohol

A depression score above 27 on the MDI was significantly less common among medical students (9.1%, n = 37 vs. 12.3%, n = 43 of business students), while controlling for sex. The effect of sex was also significant, showing a higher risk for women (Table 3). There was no association with curricular stage (data not shown).

**Table 3:** Descriptive data and logistic regressions of current depression, alcohol use, self-reported mental health problems and help-seeking^1^.

	KI Medical students				SSE Business students							
	females		males		females		males					
	%	n	%	n	%	n	%	n	**OR**_**univ**_	**CI**_**95**_	**OR**_**sex**_	**CI**_**95**_
Depression^2^	12.0	30	4.5	7	16.7	24	9.1	18	0.61*	0.37;0.98	2.31*	1.39;3.85
Harmful alcohol use^3^	10.4	26	28.0	44	25.0	36	35.4	70	0.55*	0.37;0.75	0.44*	0.33;0.63
Weekly drinking to intoxication^4^	3.6	9	12.7	20	6.3	9	19.7	39	0.58*	0.35;0.96	0.25*	0.15;0.46

No mental health problems of importance	73.3	184	84.7	133	72.9	105	86.4	171	0.97	0.67;1.40	0.46*	0.32;0.68
Have not sought help, even if needed	8.4	21	4.5	7	9.7	14	8.1	16	0.67	0.37;1.21	1.03	0.56;1.90
Have consulted student health services	6.8	17	4.5	7	2.8	4	1.0	2	3.39*	1.32;8.71	1.19	0.51:2.78
Have consulted GP	2.8	7	3.4	6	1.4	2	1.0	2	3.10	0.96;10.05	0.54	0.20;1.49
Have consulted psychiatrist/psychologist	8.0	20	2.5	4	13.2	19	3.5	7	0.53	0.28;1.01	1.02*	1.44;6.35
Have been admitted to psychiatric clinic	0.4	1	0	0	0.7	1	0	0	0.54	0.03; 8.73	-	-

Logistic regression for each variable over student group (reference category is SSE), controlling for sex (reference category is men). Adjusted odds ratios for university and as well as the covariate sex are presented. * Indicates significant group difference according to 95% confidence intervals. ^1 ^Help-seeking is reported as at any time during the course of studies. ^2^Major depression Inventory (MDI) score > 27. ^3 ^Alcohol Use Disorder Identification Test score (AUDIT) ≥ 11. ^4 ^Item 3 of the AUDIT

Harmful alcohol use (17.2% [n = 70] vs. 31.0% [n = 106]; OR 0.55, CI_95 _0.37; 0.75) and weekly drinking to intoxication (7.1%, [n = 29] v. s14.0% [n = 48]; OR 0.58, CI_95 _0.35; 0.96) were also less common among medical students than among business students. For both outcomes the adjusted effect of sex was significant, where women were less likely to score positive. For depression and weekly drinking to intoxication, no interaction effects of sex and study site were noted. For harmful alcohol use, however, there was a significant interaction effect (OR 0.49, CI_95 _0.24; 1.00, p = 0.048), indicating that being a woman and a business student may entail a particular risk. Inclusion of the interaction variable in the model only marginally affected the odds ratios of study site and sex. Curricular stage was not associated with weekly drinking to intoxication, but with harmful alcohol use. When controlling the model on harmful alcohol use for stage (OR 0.66, CI_95 _0.47;9,44), the effect of study site decreased (OR 0.23, CI_95 _0.10;0.53), whereas sex (OR 0.45, CI_95 _0.31;0.65) changed only marginally (not shown in table).

### Mental health problems in need of treatment and help-seeking

Table 3 (bottom section) shows the distribution of mental health problems in need of treatment (MHPT) and reported help-seeking. Among medical students, 77.9% (n = 317) reported no mental health problems in need of treatment, as compared to 80.7% (n = 276) among business students (not significant). Men were more likely to report no MHPT. The two student groups differed in their choice of help-seeking only with regard to student health services, which were more often sought by medical students, and there was a minor but significant tendency for women to have sought specialised help from psychologists or psychiatrists more often than men. The overall proportion of students having refrained from seeking help even if they considered they needed to, was 7.7% (n = 58), with no sex or student group difference.

### Factors associated with mental health problems in need of treatment

To assess whether MHPT was associated with burnout, study stress or alcohol habits, we performed a multivariable analysis. MHTP was entered as a dependent variable and study stress factors, burnout factors and weekly drinking to intoxication as independent variables, controlling for gender and student group, see Table 4. Being a woman, having rated above median on Low commitment, Financial concerns and Exhaustion were all independently associated with having mental health problems in need of treatment. Additional analyses, controlling for having children, a steady partner or paid employment, did not improve the model or considerably change the effect of the initially entered variables.

**Table 4:** Multivariable analysis of effect on MHPTof study stress, burnout and alcohol use, controlling for student group and sex

	OR	**95% C.I**.	p
Student group (SSE = 1, KI = 0)	1.39	0.92; 2.08	.114
Gender (women = 1)	2.01	1.32; 3.04	.001
Negative psychosocial climate^†^	1.38	0.86; 2.22	.176
Worries about future endurance/capacity^†^	1.36	0.89; 2.10	.158
Insufficient feedback^†^	0.96	0.63; 1.47	.860
Low commitment^†^	1.95	1.09; 3.51	.025
Academic workload^†^	0.73	0.47; 1.14	.167
Role conflict^†^	1.42	0.89; 2.27	.146
Financial concerns^†^	1.81	1.18; 2.80	.007
Disengagement^†^	1.44	0.91; 2.28	.121
Exhaustion^†^	2.56	1.60; 4.10	.000
Weekly drinking to intoxication	1.05	0.55; 2.01	.880

Logistic regression analysis. Cox & Snell R^2 ^0.12, Nagelkerke R^2 ^0.19

MHTP = Mental health problems in need of treatment, KI = Karolinska Institutet

SSE = Stockholm School of Econonomics

^† ^variable dichotomised by median split, scale range for all 1-4.

## Discussion

This study showed that the business students perceived more study stress and were more disengaged from their studies than the medical students. Female students of both categories were more distressed. Further, especially female business students were at risk for harmful alcohol habits. The prevalence of mental health problems in need of treatment and help-seeking did not differ between the two student groups. While medical students are often claimed to be particularly subject to high levels of distress [1,12,22,23], our present results align with some previous findings from comparative studies [10,11]. The distress repeatedly noted in medical students may thus be a phenomenon present among most university students [9] or even young people in general [7,8].

The higher levels of *study stress *and *disengagement *may apply to curricular and/or cultural differences between the two schools. The absence of graded marks at medical school in Sweden may actually serve as a buffer for study stress, as it may reduce competition and enhance peer support and thus effect the ratings of factors Negative psychosocial climate (for both sexes) and Academic workload (for women). Social support is a well-known moderator of stress, according to the demand-control model, especially so for women [24]. In addition, medical students generally stay together in the same class throughout the university career, while the business students do not have the same group cohesion. Although rated higher among business students of both sexes, the factor Insufficient feedback yielded the highest scores at both sites. Feedback is central for learning and motivation [25], thus possibly also affecting the variables Low commitment and Disengagement from studies, which were also more prominent among business students. Improving feedback strategies may be an important target for interventions, although our results do not imply that it would have a specific effect on perceived mental health.

There was no difference between the student groups with regard to exhaustion levels. One of few studies, from the US, found that business students had elevated exhaustion levels compared to "high-exhaustion" occupations, such as physicians, policemen and teachers, which were in turn associated with high coursework involvement. In contrast to the present study, this was performed during a high-stress period of final exams and no gender differences were recorded [26]. The effect of curricular stage on study stress and burnout differed between sites. For business students only Low feedback and Exhaustion differed by stage, and they were less problematic in the advanced phase; while medical students in the clinical stage reported more Worries about future endurance/capacity and Role conflict. This confirms previous findings from our group [3,27].

*Self reported depressive symptoms *were highly prevalent in the present samples, as in other studies of medical students [12,28]. The medical students did not, however, show the highest rates in our study. Independent of student group, women were more often depressed, which corresponds to the situation in the general population and Swedish university students [9,29]. In the general population, young women have comparably higher prevalence of self-rated depression than the older age groups [20]. We did not find that depression was related to stage in any of the two samples, as opposed to previous findings [30]. Medical students at the transition into clinical courses have been shown to be especially at risk for depressive reactions and stress [31], but this particular phase was not targeted by our design.

*Harmful use of alcohol *was high for men in both samples (28% at KI and 35% at SSE) and particularly notable among female business students (25%). According to a recent Swedish study, the prevalence of harmful alcohol use by the same measure was considerably lower, 7%, in Stockholm residents aged 20-34 years [20], thus close to the levels among female medical students. Among Swedish students in general, however, a high involvement of alcohol is known; female university students have their peak of alcohol consumption earlier than male students, and for both genders use of alcohol decreases during university studies [32], which was confirmed in our data. Since the alcohol habits of medical students may influence their perception of practise, where students with high alcohol intake are less inclined to counsel patients on alcohol use, [33], the recorded high prevalence of male medical students is of concern. The high alcohol consumption among business students of both sexes may be related to the different university cultures. While business students from early on engage in representation with the business sector, medical students are confronted with alcohol as a health risk. Since the study was performed, this issue has been addressed actively at the SSE, with programmes directed to enhance awareness of specific problems among female students and revisions of alcohol policies in SSE-related activities.

The prevalence of *mental health problems in need of treatment (MHPT) *did not differ between the groups and was comparable to findings from a Norwegian study of senior medical students. As opposed to our findings, however, no gender differences were found in the Norwegian sample [34]. Further, our results did not support the assumption that medical students are particularly negative to help-seeking for mental distress [16,28,35], at least not compared to business students. Medical students may have a lower threshold for consulting a professional, due to the availability of student health services on the KI campus, the like of which the SSE students do not have access to. The proportion of medical students (6.9%) that did not seek help although they thought they needed it was at level with the 6.2% among final year medical students in a Norwegian study using the same inventory [21]. Low commitment and Financial concerns as well as Exhaustion were associated with MHPT. We controlled for factors that might influence perceived stress, such as having the responsibility for children or working along with studies, but these did not add any explanatory effect. Weekly drinking to intoxication was not associated with perceived mental distress, indicating that the potentially harmful alcohol habits may not yet have rendered any negative outcomes.

There were some limitations to this study. Business students had a somewhat lower response rate. Low response rates may give a shortfall of severely distressed individuals [36] in which case, however, the differences we found would be even more pronounced. It may be argued that medical students are less willing to report symptoms of mental distress and that they also underestimate their alcohol consumption. From the discussions with the Student Associations of both universities ahead of the study, the opposite was rather expected, and the problem of under-reporting is rarely otherwise addressed in studies of medical students. Some of the measured study stress factors had low internal consistency, which may indicate some instability over the two student populations. A measure of personality in the survey would have been valuable. Although the two schools attract students of similar characteristics concerning marks and ambition, they may differ with regard to personality traits, which may affect the students' sensitivity to stress [5,37]. It is, however, possible that the study stress measure WFEC may rather reflect a personality aspect than an actual stressor. It did not differ between student groups, but women from both universities reported greater concerns about future endurance in their professional life than their male colleagues. This finding is consistent with gender differences found in studies of worry in the general population [38]. We had no data on exact year of schooling, which is known to affect distress in medical students. Nevertheless, we think the data on curricular stage may be perceived as a proxy. Finally, the generalisability of our findings is uncertain. Although the comparative sample was chosen with great deliberation and considered appropriate, the influence of cultural, curricular and local factors on the findings cannot be ruled out. The lack of comparative studies in the field is striking; for the sake of generalisability, future research should preferably include national and multicentre studies.

## Conclusions

We found that medical students were less affected with mental distress and harmful alcohol use than business students. The frequently stated high levels of stress among medical students may thus not be exceptional compared to other student populations. A higher awareness of a healthy lifestyle, the absence of stress from graded marks and a more socially cohesive structure of medical education may act as beneficial factors. Screening for mental distress at local universities to evaluate the need for preventive measures and improved availability of local student health services would be valuable.

## List of abbreviations

HESI: Higher Education Stress Inventory; WFEC: Worries about Future Endurance/Capacity; OLBI: Oldenburg Burnout Inventory; AUDIT: Alcohol Use Disorder Identification Test; IQR: Interquartile Range; SSE: Stockholm School of Economics; KI: Karolinska Institutet; MHTP: Mental Health Problems in need of Treatment; OSCE: Objective Structured Clinical Examination

## Competing interests

Marie Dahlin and Bo Runeson both teach at Karolinska Institutet medical school and funding was received from the Board of Education of Karolinska Institutet, which has had no influence on the design, analyses or results of the study.

## Authors' contributions

BR arranged funding and planned the study. CN took part in planning the study, carried out the data collection and initial statistical analyses and wrote a first draft of the manuscript. ES interpreted data. MD planned the study, performed statistical analyses and wrote the manuscript. All authors took part in data interpretation, revision of the manuscript and have read and approved the final manuscript.

## Authors' information

BR is Professor of Psychiatry at KI, his main research field is within Suicidology. MD is Senior Lecturer in Psychiatry at KI, her main research field is within Student Health. CN was a medical student at KI at the time of data collection, currently doing her preregistration year. ES is an MD, with a current position as resident in Obstetrics/Gynaecology.

## Pre-publication history

The pre-publication history for this paper can be accessed here:

http://www.biomedcentral.com/1472-6920/11/92/prepub
